# Quality of Life with Macular Degeneration Is Not as Dark as It May Seem: Patients’ Perceptions of the MacDQoL Questionnaire

**DOI:** 10.3390/jcm4091841

**Published:** 2015-09-22

**Authors:** Lisa M. Ord, JoAnne Wright, Margaret M. DeAngelis, Michael Feehan

**Affiliations:** 1 School of Medicine, University of Utah, John A. Moran Eye Center, Salt Lake City, UT 84132, USA; E-Mail: margaret.deangelis@utah.edu; 2 Division of Occupational Therapy, College of Health, University of Utah, Salt Lake City, UT 84108, USA; E-Mail: joanne@xmission.com; 3 School of Health Sciences, Salt Lake Community College, West Jordan, UT 84088, USA; 4 College of Pharmacy, University of Utah, Salt Lake City, UT 84112, USA; E-Mail: mike.feehan@utah.edu

**Keywords:** age-related macular degeneration, quality of life, MacDQoL Questionnaire, visual impairment

## Abstract

To determine the perceived relevance and value of an individualized measure of the impact of macular degeneration on quality of life (QoL) for elderly people with Age-Related Macular Degeneration (AMD) in the USA, through the assessment of the suitability of the measure’s domains and by gaining a deeper insight into the impact of AMD on patients’ QoL vis-á-vis these domains, community-dwelling older adults in the metropolitan Salt Lake City, Utah area were interviewed using the macular degeneration on quality of life (MacDQoL) instrument. Participants felt that the MacDQoL was a relevant instrument for use in this US study population, though it could be improved by adding items pertaining to transportation, and independent driving, in particular, as an important QoL indicator. The emerging theme from analysis of the respondent’s commentary was that, in spite of AMD, these respondents were committed to engage in, and enjoy life. This is an important concept for clinicians and those who offer support programs to integrate into their care planning and reinforce in messaging to patients with the condition.

## 1. Introduction

Age-Related macular degeneration (AMD) is the leading cause of blindness in the United States and the third leading cause worldwide [1,2]. The National Eye Institute predicts that over 5.4 million people in the United States will have AMD by the year 2050 [1]. There are two types of age-related macular degeneration: dry (atrophic; nonexudative) and wet (neovascular; exudative). Approximately 85% to 90% of AMD cases are of the dry type which has a slow progression of macula photoreceptor cell deterioration and retinal pigment epithelial (RPE) hypo and/or hyper pigmentation. The wet type can have a rapid onset caused by the growth and leakage of abnormal vasculature of the retina [3] and accounts for approximately 90% of blindness related to macular degeneration [4]. Although advances in the understanding of macular degeneration have led to better treatments [5] in the United States people reporting a visual disability increases dramatically with age, 15% of adults aged 45 to 65 have vision impairment compared to 26% of adults over the age of 75 years [6].

The severity of vision loss should be evaluated by a combination of objective measures including acuity, contrast sensitivity, and, most importantly, functionality in everyday vision-related tasks. However, the experience and impact of vision loss on quality of life (QoL) remains subjective. The impact of vision loss is reflected by the patient’s perspective [7]. Individuals with similar clinical features may function very differently, experience different levels of impairment [8], and report disparate values of perceived QoL, across the physical, mental, emotional and social domains of functioning. QoL is a fairly recent focus of research with approximately 87% of all research using structured assessment tools conducted in the last 20 years and 50% in the last decade [7]. Generic health-related quality of life measures provide a summary of quality of life aspects which relate to an individual’s overall physical health, mental, and social well-being. Condition or disease-specific quality of life measures have a more narrow focus on life aspects that are specifically affected by the disease or health condition [9]; vision-specific quality of life measures are more sensitive to changes in an individual’s functioning which translate into perceived quality of life [10]. Studies that analyze the utility of generic *versus* condition-specific health-related quality of life measures based on construct and convergence validity, responsiveness and reliability find that as visual impairment worsened, generic health-related measures also worsened on utility values [11,12]. In a review of literature on QoL in AMD, Yuzawa and colleagues [13] found that the impact of AMD on QoL is severity-dependent and required disease-specific QoL measures for adequate sensitivity.

Few instruments specifically gauge the QoL of individuals suffering eye diseases such as AMD. One such is an individualized measure of the impact of macular degeneration on quality of life, the MacDQoL [14] developed in the UK, designed to measure QoL in individuals suffering from AMD, overall and in specific domains of functioning. The questionnaire assesses the impact of vision impairment on a particular life domain, as well as the importance an individual places on that domain.

The central aim of the study was to qualitatively gauge the utility of the MacDQoL for elderly people with AMD in the USA. This aim is satisfied through three areas of structured inquiry: (a) the assessment of the perceived relevance of the MacDQoL; (b) determining if the domains assessed were suitable or needed to be modified for this population; and (c) gaining a deeper insight into the impact of AMD on patients’ QoL vis-á-vis the domains of the MacDQoL.

## 2. Methods

The protocol was reviewed and approved by the Institutional Review Board at the University of Utah (Salt Lake City, Utah, USA) and conforms to the tenets of the Declaration of Helsinki. Eligible patients were enrolled in this study after they gave informed consent in person. Participants were 27 community-dwelling adults (six men and 21 women), over the age of 64 years recruited from the John A. Moran Eye Center Patient Support Program, University of Utah College of Health Life Skills Clinic, and senior apartment complexes along the Wasatch Front. Interviews were conducted over a period of 14 weeks. All participants had a diagnosis of AMD from an ophthalmologist in the Salt Lake City area for more than one year and were able to read (ability, should magnification be great enough) and speak English. People who had a dramatic drop of vision or experienced negative health or life events in the previous six months were excluded from the study to avoid the influence of potential emotional impact from recent events, and to reflect the impact of vision impairment rather than other life events. Participants completed a demographic form, visual functioning was tested using the Smith-Kettlewell Reading Test, and the MacDQoL was then administered by having the interviewer read the questions out loud and recording the participant’s answers. Participants were encouraged to comment throughout the administration of the MacDQoL, and their comments were audio-recorded. A semi-structured interview focused on the participant’s perception of how accurately the items on the questionnaire captured the different factors that affect their QoL was conducted and audio-recorded.

### 2.1. Instruments

#### 2.1.1. The MacDQoL

The MacDQoL [14,15] was designed to measure overall quality of life in individuals with AMD as well as the impact of AMD on quality of specific life aspects or domains. The MacDQoL assesses the impact of vision impairment on a particular life domain *as well as* the importance an individual places on that domain. Participants respond to 26 questions, covering 22 domain-specific items, about their perception of the impact of AMD on life aspects, scored from −3 (maximum negative impact) to +1 (maximum positive impact) and the importance of each aspect from 0 (not at all important) to 3 (very important). A weighted impact score (WI) is calculated for each domain by multiplying the impact score by the importance score (range: −9 maximum negative impact to +3 maximum positive impact). For example, in the household tasks domain a participant answers that if she did not have macular degeneration she could handle her household tasks much better (score −2). Then she answers that handling her household tasks is very important to her (score 3). The WI for the participant’s household tasks domain is 6 (−2 × 3 = −6). An overall average weighted impact (AWI) score is calculated by summing each domain WI, then dividing by the number of answered domains. Table 1 lists the 22 domain-specific items on the MacDQoL.

**Table 1:** Macular degeneration on quality of life (MacDQoL) domain-specific items.

No.	Domain
1	Household tasks
2	Personal affairs (letters, bills, *etc.*)
3	Shopping
4	Closest personal relationship
5	Family life
6	Friendships and social life
7	Physical appearance (clothes and grooming)
8	Physical activity
9	Getting out and about (e.g., car, foot, bus, or train)
10	Travel
11	Leisure activities (e.g., reading, TV, radio, gardening, hobbies)
12	Self-confidence
13	Motivation
14	People or society reactions to me
15	Feelings about the future
16	Financial situation
17	Independence
18	Doing for others
19	Mishaps or losing things
20	Enjoying meals
21	Time it takes to do things
22	Enjoying nature

#### 2.1.2. Smith-Kettlewell Reading Test (SK Read)

Reading is very often the first aspect of functioning in which patients notice a visual impairment. The SK Read [16] was administered to measure macular function and the presence of scotomas. Blocks of text are printed in high contrast black on white and range in text size from 8 M units to 4 M units (1 M = smallest newsprint). Each block contains 60 characters, including spaces, with a combination of single letters and common words. Participants are instructed to read the text aloud. Reading performance is measured in the average number of seconds needed to read each block of text and the average errors per block of 60 characters.

#### 2.1.3. Semi-Structured Interview

Following the administration of the MacDQoL survey and SK Read tests, participants were interviewed with five questions, which were then probed on for further detail by masters-level students of the University of Utah, Division of Occupational Therapy. These were: (1) What parts of the survey relate most to your experience with macular degeneration? (2) What parts of the survey relate the least? (3) How well does the survey reflect your experience of living with macular degeneration? (4) What areas of life does the survey not cover? (5) What have you found that helps you deal with the effects of macular degeneration?

### 2.2. Thematic Analysis Methodology

Thematic analysis is a method in which patterns in the data are identified and categorized according to theme, topic, or idea. Thematic analysis is not rooted in any particular epistemology, allowing a more accessible form of analysis [17]. In our analysis, we take a phenomenological and critical realism approach in viewing the data as the individual’s experience of reality and the meaning that the individual makes of that experience. Comments were analyzed by the prevalence of themes either by the number of instances in which the theme arose in the statements across all interviews and questionnaires or if the statement demonstrated an important point in the experience of the individual or individuals.

Audio transcripts of the MacDQoL and semi-structured interview were reviewed by three of the authors (Lisa M. Ord, JoAnne Wright, Michael Feehan) for prominent themes that captured living with AMD and its associated impact on their QoL. Given the restricted nature of the questions the volume of data were such that themes could be identified and coded without recourse to structured qualitative analysis software (e.g., NVivo, Version 10, 2012, QSR International Pty Ltd., Doncaster, Victoria, Australia.)

## 3. Results

Results are reported for 26 respondents. One participant was eliminated from analyses as he did not have any noticeable vision loss at the time of the interview.

MacDQoL overall AWI scores ranged between −1.04 (least impact on QoL) and −8.40 (greatest impact on QoL) with a median score of −3.15. Demographics are reported in Table 2.

**Table 2:** Demographics and Smith-Kettlewell Reading Test (SK Read) scores.

	Total
Gender	26 (100%)
*Male*	5 (19%)
*Female*	21 (81%)
Mean Age	82.0 (7.9 SD)
Marital Status	
*Widowed*	14 (54%)
*Married*	8 (31%)
*Divorced/Single*	4 (15%)
Living Situation	
*Alone*	18 (69%)
*With Spouse*	7 (27%)
*With Children*	1 (4%)
Education	
*High School*	4 (15%)
*Some College to BA/BS*	16 (62%)
*Master’s to Doctorate*	5 (19%)
*Other*	1 (4%)
Mean Years Diagnosed	15.9 (11.4 SD)
SK Read Mean (seconds/block)	39.1 (39.2 SD)
SK Read Mean (mistakes/block)	2.6 (3.8 SD)

SD, standard deviation; BA, Bachelor of Arts; BS, Bachelor of Science.

### 3.1. MacDQoL Questionnaire

Based on mean WI scores, Table 3 ranks the most impacted life domains. *Independence* ranks in the top domain that people report as “very important” and in which their life would be “much better” without macular degeneration.

**Table 3:** Top 10 most negatively impacted MacDQoL domains.

Domain	All Participants Rank	Weighted Impact	SD
Independence	1	−5.88	2.89
Personal Affairs	2	−5.73	3.89
Doing for Others	3	−5.42	3.23
Leisure Activities and Hobbies	4	−4.96	3.17
Family Life	5	−4.85	3.48
Do Physically	6	−4.77	2.94
Enjoy Nature	7	−4.73	3.48
Get Out and About	8	−4.69	3.25
Household Tasks	9	−4.54	2.67
Friends and Social	10	−4.38	3.19

SD, standard deviation.

### 3.2. Perceived Relevance of the MacDQoL

In response to the semi-structured interview questions concerning the relevance of the MacDQoL, most participants felt that the life domains presented in the MacDQoL were very relevant to their own life.

It’s interesting that these questions are asking the things that really are important to me, the things that I’ve always depended on that I find I can’t do anymore. Just right on the spot.

*—85-year-old woman*

That’s a pretty good questionnaire.

*—84-year-old woman*

I thought it was comprehensive and very thorough.

*—84-year-old man*

All the questions are very, what can I say, very … to the point of what, how it affects us. I mean the questions you ask are all things that we encounter.

*—88-year-old woman*

The ability to independently drive a car was identified by participants as the one key domain that the survey does not address. This may reflect differences between USA and UK elderly population cultures. Fifteen participants (56%) reported that no longer being able to personally drive had a negative impact on their quality of life.

You know, you can do anything if you want to whether you’re blind or not. Uh, except drive.

*—64-year-old woman*

My whole problem centers around driving. I’ve got friends that live in [town] and it’s just I used to go see them all the time and they work and it’s too hard. By the time they get off work, drive out here, get me, drive me back, you know. So you lose a lot of friendships. And to be able to take the kids to dance class or to be able to take the kids to karate or to be able to take the grandkids to the dentist or the doctor, go pick up this or go do that for my girls or the neighbor or whatever. If I could drive, yeah, it would make things a whole lot better. … like I said, it’s just the driving that drives me crazy. Because I don’t even mind not being able to see.

*—64 year-old woman*

And once you take your driving away from you you’ve given up your independence if you can’t drive a car anymore.

*—85-year-old woman*

I can type, I’ve always typed by touch but that and driving and recognizing people, in a way that’s a physical thing. Those are things that I can’t do anymore or can’t do them well and so that’s where my macular degeneration has profound impact on me.

*—84-year-old man*

Participants identified the questionnaire domains most related to his or her experience as, in order of most endorsed, (1) “all” domains (2) leisure activities and hobbies (3) getting out and about and (4) independence. Leisure activities, getting out, and independence all ranked in the top ten most negatively impacted domains when responses for all participants were analyzed together. Only the interactions with others and enjoying meals domains were noted to be least relative to participants.

The majority of participants (88%) identified one or more of four things that helped them deal with vision loss from AMD: assistive devices or functional changes to environment, good social support, acceptance and positive attitude, and continuing to do things they love and engage in life.

### 3.3. Impact of AMD on QoL Vis-á-Vis Domains of the MacDQoL

Although “frustration” was echoed in all domains on the MacDQoL, the emerging theme from analysis of the respondent’s commentary was that life may not be as bleak as a non-sufferer may expect, and that these respondents were committed to engage in, and enjoy life. They may make lifestyle modifications to adapt to their vision loss, but still maintain a positive view of life.

How has macular degeneration ruined my life? How has macular degeneration ruined my meals, ruined my shopping, ruined my enjoyment of nature? Well, it hasn’t really.

*—72-year-old woman*

Oh, I’m sure [life] would be much better, but you just have to deal with what you have to do.

*—75-year-old man*

To illustrate this theme and the perseverance of those with AMD to maintain engagement, the following case is helpful. Mrs. Z. (aged 85) had the most impacted QoL of the 26 respondents (total average weighted impact score = −8.40 out of −9.0). Even reporting a greater impact of AMD on quality of life, she maintains positivity.

… I love to learn, I love to see things and so I’ve really, really had to talk to myself. A lot of people are worse off than I am.

Engaging in social activities she used to enjoy when fully sighted is an important factor in Mrs. Z’s life, and even when she does not feel comfortable she still engages in social activities.

[I’ve] always had a book club and I’ve read many, many books but now when I go to the book club I’ll listen but I’m not comfortable there anymore. So I really have to push myself to go because it’s a good thing for me.

Despite having AMD she still goes out and ensures she maintains a good appearance by adapting her makeup and hair routine (enlisting husband):

I haven’t always worn a lot of makeup, only when I go out, but I’ve always put lipstick and mascara on and of course I can put the lipstick on because I’ve done it so many years, just blindly putting it on. But that makes a difference when I go out.

Others also engage in keeping up appearances despite AMD, persevering through frustration to maintain a positive self-esteem.

I like to put nail polish on my fingernails but I can’t find the hole in the bottle. I can get the brush out but I can’t get it back in because I am going all around it and I’ve got fingernail polish all over the bottle.

*—Ms. Y, 82-year-old woman*

Although reporting that her quality of life is very poor, Mrs. Z continues to do the things she loves, like walk to the grocery store, knit, or go out for dinner:

When we go out for dinner it is a hard thing for me to use my fork to pick up salad. So I finally decided that I’d just have soup. I’ll have some kind of fantastic soup. I get along better with that than anything else.

Mrs. Z has learned to adapt her way of doing things and has learned that she can ask for help when needed. She doesn’t sit at home and mope, instead, even though things aren’t as easy or as enjoyable as they once were, she continues to engage in life.

Mrs. C, age 89 years, attributes much of her continued engagement in life to her positive personality:

I’ve always been a positive personality … if [society at large] doesn’t react, they don’t understand. I don’t let it bother me. For the most part, they’re very kind.

If Mrs. Z scored the lowest on the MacDQoL yet is still engaging in life, what kind of comments were made by the person with the highest MacDQoL score indicating AMD made the least amount of impact on quality of life? Mr. A is a 75-year-old married man who states he has trouble making out features in faces but reports that everything simply takes longer:

I pretty much do everything that I’ve done before [but] I have a lot of trouble doing the stuff … I would like to do a lot more things than I do, and I do quite a few of the things that I’ve always done, but it just takes forever.

Other participants were not as optimistic about their life with AMD:

There are days when I get really frustrated. And in my house I call it being pissy. It’s best not to talk to me.

*—Mrs. R, 72-year-old woman*

With the help that I have I get by but it's a challenge, a big challenge, you know. It gives you a terrible feeling, one of insecurity. Then there’s the other part of making more work for [wife].In the past I took care of the bills and the bank account and all of those things. I did the driving, kept the car up and all that. I know what lies ahead and I know what’s happening now, but I would like to be able to handle it maturely and without being a problem to other persons. It’s always a big concern.

*—Mr. W, 84 year-old man*

For me, getting out and about is very important. I get a terrible depression if I can’t get out. I’ve become very fearful about crowds and [public transportation].

*—Mrs. R, 72-year old woman*

## 4. Discussion

This study addressed the aim of determining the utility of the MacDQoL in our elderly US group of respondents. The MacDQoL appears relevant to respondents’ experience and the domains are generally suitable. The measure could be enhanced with an explicit focus on personal driving ability. Across the domains measured by the MacDQoL, our respondents maintained a positive response to the impact of AMD on the quality of life.

The MacDQoL can open a dialog about visual impairment and how an individual’s life is affected by vision loss. It appears to be a suitable instrument for use in this patient population, though it could be improved by including a transportation domain. Although aspects of driving are included in MacDQoL domain “getting out and about”, many participants either commented that driving was an area not explicitly covered by the questionnaire or they had repeatedly commented on not being able to independently drive as one of the most frustrating aspects of having AMD. This may indicate that driving may represent more to Americans. Driving in the US has historically been equated with freedom, free agency, and the unrestricted liberty to explore the “open road” [18]. Therefore, the addition of an “Independent Transportation” domain, specifically to include driving, could be added to increase the salience of questionnaire for US populations. Losing the ability to drive may be a quality of life identifier even more specific to living in the Western US, where driving may be more associated with independence, rather than living in Manhattan, NY. The difference in the SK Read mean time between participants scoring a lower average weighted impact of AMD on QoL and a higher average weighted impact of AMD on QoL on the MacDQoL is not statistically significant; however, it appears that people who report a greater negative impact of AMD on quality of life may have poorer visual acuity or more severe scotomas. Knowing their visual difficulties are severe, affected individuals may take more time to make out the test letters and words and therefore actually make fewer mistakes per reading block. This may also indicate that QoL may not be dependent solely on useful vision.

Ratings of whether performance on specific QoL indicators would be better without AMD may compel the individual to compare their life before vision loss to his or her present functioning without factoring in the age difference (life with a younger more healthy body), as evidenced by statements of comparison such as:

Of course much better because you can’t do quite the same things you always did …

*—92-year-old woman*

[Handling personal affairs would be] very much better. This is what I can’t do anymore.

*—85-year-old man*

I cannot pull the whiskers on my chin and all the personal things that I used to do I can’t do now because I can’t see that well.

*—85-year-old woman*

While a weighted impact score for each domain is a helpful way to examine how AMD affects individuals, it has come under some scrutiny for using a multiplicative rating scale (using two measurements to create a new characteristic) [19].

Critically however, as these participants have pointed out, struggling to do things or giving up old hobbies or interests does not mean that the elderly individual is no longer engaging in life or feeling that his or her overall quality of life is inevitably poor. Despite the highly disabling condition, sufferers of AMD continue to engage, adapt to compensate for their vision loss, and while they may feel frustrated, they are not in any sense giving up on life activities. This is an important concept that clinicians and those who offer support programs should integrate into their care planning and reinforce in messaging to patients with the condition. Patient education materials and communications should emphasize the positive life engagement of those with AMD, even those who may at first seem to be most impacted (when measured by instruments such as the MacDQoL).

Limitations of this study include a small sample size and the relatively narrow breadth of inquiry focused purely on the domains of the MacDQoL. Nevertheless, we do get some clear insights on the usefulness of the MacDQoL to open a dialog and as a QoL tool.

It is the intent of the authors to use the MacDQoL in a more comprehensive longitudinal qualitative and quantitative evaluation of patients who vary in their resilience in the face of the course of their AMD, with particular regard to maintaining life function and sound mental health.

## References

[1] National Eye Institute Age-Related Macular Degeneration. https://www.nei.nih.gov/eyedata/amd.asp.

[2] World Health Organization Global Data on Visual Impairments 2010. http://www.who.int/blindness/GLOBALDATAFINALforweb.pdf?ua=1.

[3] Yonekawa Y., Kim I.K. (2015). Clinical characteristics and current treatment of age-related macular degeneration. Cold Spring Harbor Perspect. Med..

[4] Ferris F.L., Fine S.L., Hyman L. (1984). Age-related macular degeneration and blindness due to neovascular maculopathy. Arch. Ophthalmol..

[5] Yonekawa Y., Miller J.W., Kim I.K. (2015). Age-related macular degeneration: Advances in management and diagnosis. J. Clin. Med..

[6] Leonard R. Statistics on vision impairment: a resource manual. Arlene R. Gordon Research Institute of Lighthouse International. http://www.gesta.org/estudos/statistics0402.pdf.

[7] Fletcher D.C., Schuchard R.A. (2006). Visual function in patients with choroidal neovascularization resulting from age-related macular degeneration: The importance of looking beyond visual acuity. Optom. Vis. Sci..

[8] Crossland M.D., Culham L.E., Rubin G.S. (2005). Predicting reading fluency in patients with macular disease. Optom. Vis. Sci..

[9] Cunningham S.J., Garratt A.M., Hunt N.P. (2000). Development of a condition-specific quality of life measure for patients with dentofacial deformity: I. Reliability of the instrument. Community Dent. Oral Epidemiol..

[10] Scott I.U., Smiddy W.E., Schiffman J., Feuer W., Pappas C.J. (1999). Quality of life of low-vision patients and the impact of low-vision services. Am. J. Ophthalmol..

[11] Longworth L., Yang Y., Young T., Mulhern B., Hernández Alava M., Mukuria C., Rowen D., Tosh J., Tsuchiya A., Evans P. (2014). Use of generic and condition-specific measures of health-related quality of life in NICE decision-making: A systematic review, statistical modelling and survey. Health Technol. Assessment.

[12] Tosh J., Brazier J., Evans P., Longworth L. (2012). A review of generic preference-based measures of health-related quality of life in visual disorders. Value Health.

[13] Yuzawa M., Fujita K., Tanaka E., Wang E.C.Y. (2013). Assessing quality of life in the treatment of patients with age-related macular degeneration: Clinical research findings and recommendations for clinical practice. Clin. Ophthalmol..

[14] Mitchell J., Bradley C. (2004). Design of an individualized measure of the impact of macular disease on quality of life (the MacDQoL). Qual. Life Res..

[15] Mitchell J., Wolffsohn J.S., Woodcock A., Anderson S.J., McMillan C.V., Ffytche T., Rubinstein M., Amoaku W., Bradley C. (2005). Psychometric evaluation of the MacDQoL individualised measure of the impact of macular degeneration on quality of life. Health Qual. Life Outcomes.

[16] Fletcher D.C., Nair U., MacKeben M., Schuchard R.A., Schneck M.E., Watson G. (2008). Smith-Kettlewell Reading Test (SK Read).

[17] Braun V., Clark V. (2006). Using thematic analysis in psychology. Qual. Res. Psychol..

[18] Seiler C. (2008). Republic of drivers: A cultural history of automobility in America.

[19] Finger R.P., Fenwick E., Pesudovs K., Marella M., Lamoureux E.L., Holz F.G. (2012). Rasch analysis reveals problems with the multiplicative scoring in the Macular Disease Quality of Life Questionnaire. Ophthalmology.

